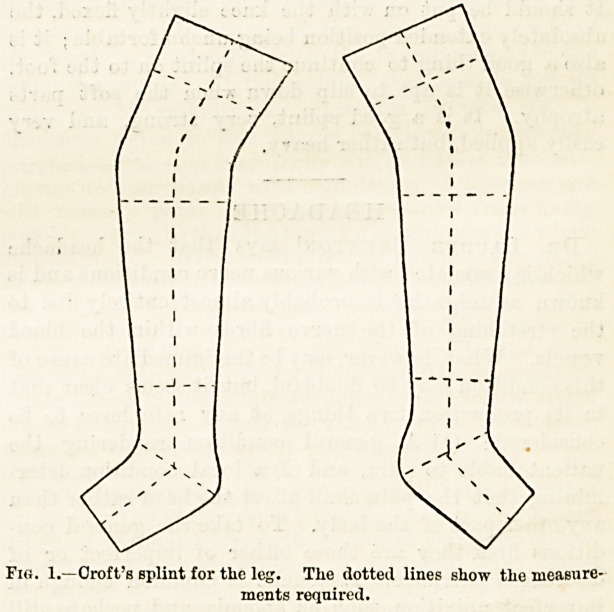# Some Plaster of Paris Splints

**Published:** 1899-11-11

**Authors:** L. A. Bidwell

**Affiliations:** Senior Assistant Surgeon to the Hospital, Dean of the Postgraduate College.


### Hospital Clinics and Medical Progress.

### SOME PLASTER OF PARIS SPLINTS.

A Postgraduate Lecture, delivered at the West London Hospital by L. A. Bidwell, F.R.C.S., Senior Assistant Surgeon to the Hospital, Dean of the Postgraduate College.

Plaster of Paris commends itself to us on account of the ease with which it can always be obtained, the rapidity with which it sets, and by the strength of the splint formed. Its main disadvantage is its weight, and, when a circular plaster case is applied, its lack of expansion, should the limb become swollen. It is impossible in one lecture to describe all the various forms in which plaster of Paris is used, so I will specially draw your attention to four useful splints. There are three principal ways in which plaster splints can be applied: (1) The application of plaster of Paris bandages, (2) Croft's method, and (3) the application of strips of house-flannel soaked in plaster of Paris solution. The first method is probably well known to you all. The bandages are prepared by rubbing in plaster of Paris before they are rolled; they require to be soaked in water for some minutes before use. The best material for these bandages is a stiff muslin called crinolette; the ordinary open wove absorbent bandage, however, answers very well. I consider these bandages quite unsuitable in the treatment of fractures, and I reserve their use for the application of Sayre's jackets, and for fixing the foot and toes after wrenching or tenotomy. The great objection to splints formed in this way is, that since the limb is completely surrounded with a rigid case no swelling of it is possible, and so if one should be applied immediately after a fracture great pain results, and even gangrene of the limb has been known to occur. Another great disadvantage of this kind of splint is the difficulty experienced in taking it off; it requires either to be sawn through, or divided with Seutin's pliers, but it is possible to remove the splint by soaking the limb for some time in hot water, when it will be found possible to unwind the bandages. The second method bears the name of its introducer, Mr. Croft, one of the past surgeons of St. Thomas's Hospital. It is a modification of the Bavarian splint, which was used in the Franco-Prussian war. It is made of house or Bavarian flannel, in two halves. Each half consists of two pieces of flannel, which are fitted accurately to the limb, and are cut after careful measurements; their fit tested on the sound limb; this is done for two reasons, first to save pain in moving the injured part, and secondly in order that the splints may fit when the swelling has subsided. The two pieces which are to lie next to the skin, and are of the shape shown in Fig. 1, are laid out on the table with their inner surfaces downwards; the other two pieces are soaked in a solution of plaster of Paris, of the thickness of thin cream, and are used as sponges to rub the plaster into the outer surfaces of the inner parts of the splints; they are then spread out on the top of the other pieces, and the splint is ready to be applied. This is done directly to the limb without any wool or bandage underneath, since there is no plaster on the inner surface of the splint, and consequently it will not stick. When moulded to the limb it is fixed with an absorbent bandage. We will illustrate this by putting up the leg for a supposed fracture of the tibia and fibula; the splint must, of course, fix both the knee and the ankle joints. The measurements required are the circumferences of the thigh near the knee, of the leg just below the knee and above the ankle, round the heel and ankle, and round the foot near the toes; the length is taken from the top of the splint to the internal malleolus, and thence to the ball of the great toe. These can be taken with a strip of the flannel, and notches should be made to indicate the position of the knee and of the ankle. The pieces used for taking the circumferences are divided in half, and all the measuring strips are then placed on a large piece of Bavarian flannel and the splint cut out; after trying this on the sound limb, three others are cut out to match. Before applying a shaving is cut off each half, so as to ensure an interval of half an inch between the two halves of the splint both in front and behind when the parts are moulded on. In putting

Fig. 1.—Croft's splint for the leg. The dotted lines show the measurements required.

on care must be taken to keep the ankle well flexed, and a slight degree of flexion of the knee joint also is desirable. This method of applying a plaster splint is quite satisfactory, but it is a little troublesome, since it requires careful measurements and accurate adjustment in order to ensure a good fit, and it cannot be applied without some assistance. It is easily removed by cutting the bandage in the interval between the two lateral halves of the splint; moreover, these two pieces can be repadded and reapplied, and of course fit perfectly. In case of any swelling of the limb no strangulation will occur, since there is an interval both at the front and back of the plaster which is covered only by the open wove bandage, a material which allows a considerable amount of "give." We therefore need have no hesitation in applying one of these splints to a fractured leg immediately after the accident. When all the swelling has subsided, after a fracture, it will be necessary to remove the splint, and, after padding, to reapply it. The two halves can be firmly fixed by using a calico bandage, over which a solution of gum is painted; in the case of a splint on the thigh or leg of a child some melted hard paraffin can be used in a similar way, and makes an elegant casing, which further prevents the plaster from being rotted by urine. The third method of applying a plaster splint is more simple than the last discribed, and needs no preparation, Six or eight strips of house flannel are cut or torn the required length and about two inches wide; these are soaked in a solution of plaster of Paris the consistency of thin cream; a flannel bandage is applied, and over this the plaster strips are applied lengthwise round the limb, slightly overlapping one another, but leaving an interval of half an inch along the whole length of the splint on one side; an open wove bandage keeps the strips in place. To remove this, all that is necessary is to cut the bandages (open wove and flannel) at the line where the plaster is deficient, and then to open up the splint. It cannot be reapplied in the satisfactory manner in which the Croft's splint can be. The splint is useful in a case of fracture of the patella, or in disease of the knee joint. It should be put on with the knee slightly flexed, the absolutely extended position being uncomfortable; it is also a good thing to continue the splint on to the foot, otherwise it is apt to slip down when the soft parts atrophy. It is a good splint, very strong, and very easily applied, but rather heavy.

## Figures and Tables

**Fig. 1. f1:**